# Gender Differences in Predictors of Physical Functioning Limitations Among the Elderly Population in Serbia: A Population-Based Modeling Study

**DOI:** 10.3390/medicina61030508

**Published:** 2025-03-16

**Authors:** Dejan Nikolic, Natasa Mujovic, Milena Santric-Milicevic, Sindi Mitrovic, Nevena Krstic, Ana Radic, Diana Radovic, Ardak Nurbakyt, Dinara Sukenova, Milena Kostadinovic

**Affiliations:** 1 Faculty of Medicine, University of Belgrade, 11000 Belgrade, Serbia; natasa.mujovic@med.bg.ac.rs (N.M.); sindi.mitrovic@med.bg.ac.rs (S.M.); nevena.krstic@med.bg.ac.rs (N.K.); 2 Department of Physical Medicine and Rehabilitation, University Children’s Hospital, 11000 Belgrade, Serbia; 3 University Clinical Center of Serbia, 11000 Belgrade, Serbia; milena8250@gmail.com; 4 School of Public Health, Faculty of Medicine, University of Belgrade, 11000 Belgrade, Serbia; milena.santric-milicevic@med.bg.ac.rs; 5 Clinic for Rehabilitation “Dr Miroslav Zotovic”, 11000 Belgrade, Serbia; ana.radic@rehabilitacija.rs; 6 Institute of Rehabilitation, 11000 Belgrade, Serbia; diana.radovic.bg@gmail.com; 7 Department of Public Health, Asfendiyarov Kazakh National Medical University, Almaty 050000, Kazakhstan; a.nurbakyt@kaznmu.kz (A.N.); sukenova.d@kaznmu.kz (D.S.)

**Keywords:** gender, elderly, general health, chronic diseases and conditions, functioning

## Abstract

*Background and Objective:* Active aging is influenced by various factors, including chronic diseases, multimorbidity, functional limitations, and disabilities. The presence of these factors might lead to greater dependence on caregivers and could present potential barriers to community engagement. Physical functioning might be considered as one of the vital components for healthier aging experience promotion and support in elderly people. This study aimed to assess self-perceived general health and related health characteristics among the elderly population in Serbia, with a focus on varying degrees of functional limitations, as well as to analyze the predictors of physical functioning limitations in relation to gender. *Materials and Methods:* This population-based modeling study included a representative sample of 3540 elderly individuals aged above 65 years from Serbia. We employed a dual approach to model the four distinct difficulty levels related to the two groups of limitations of physical functioning (PF1 and PF2) for both genders. The PF1 focused on walking half a kilometer on level ground without the assistance of any mobility aids, and the PF2 navigated a set of 12 steps of ascent and descent: Model 1: inability to execute PF1, Model 2: some/a lot of difficulty in PF1, Model 3: inability to execute PF2, and Model 4: some/a lot of difficulty in PF2. Further variables were evaluated: self-perceived general health, long-lasting health problems, and chronic diseases/chronic conditions. Logistic regression analysis was performed to assess predictors of physical functioning. The models’ performance was presented. *Results:* Significant predictors were as follows: self-perceived general health (Model 1 (male OR: 8.639; female OR: 3.569); Model 2 (male OR: 2.759; female OR: 2.277); Model 3 (male OR: 24.290; female OR: 5.090); Model 4 (male OR: 3.256; female OR: 2.152)); long-lasting general health (Model 1 (female OR: 2.867); Model 3 (female OR: 3.602)); pulmonary diseases (Model 2 (male OR: 2.036); Model 4 (male OR: 1.976; female OR: 1.756)); musculoskeletal diseases (Model 1 (female OR: 1.537); Model 2 (male OR: 1.397; female OR: 1.410); Model 3 (male OR: 1.954; female OR: 1.739); Model 4 (male OR: 1.531; female OR: 1.483)); and other chronic diseases (Model 3 (male OR: 2.215)). *Conclusions:* Bad self-perceived general health and pulmonary and musculoskeletal diseases were predictors of functional disability in both genders of elderly individuals, while long-lasting health problems were predictors of functional disability in females and other chronic diseases were predictors in elderly males.

## 1. Introduction

In recent decades, the global demographic shift toward an aging population has prompted health authorities to prioritize active and healthy aging within their health policies [1]. Active aging is influenced by various factors, including chronic diseases, multimorbidity, functional limitations, and disabilities. The presence of these factors might lead to a greater dependence on caregivers and could present potential barriers to community engagement. Therefore, physical functioning might be considered as one of the vital components for healthier aging experience promotion and support in the elderly.

It is estimated that more than 46% of individuals aged 60 years and above have disabilities, and it is projected that between the period of 2015–2030, the number of people aged 60 years and above will increase by 56% [2]. Data from 2019 Eurostat stated that 49.7% of European Union (EU) elderly reported moderate or severe difficulties considering personal care and household activities, with females more affected than males in EU (57.1% and 39.9%, respectively) [3]. It is worth mentioning that disability in elderly increases the risk of death, particularly in people with chronic diseases [4].

Despite the fact that chronic diseases have lower rates in aging women compared to male counterparts, it is reported that females have increased rates of functional limitations and disability [5]. Numerous factors might contribute to such facts including more prevalent musculoskeletal conditions in females [6,7], lower pain tolerance and thresholds, as well as more intensive pain experience in females [7], and higher prevalence of multimorbidity in females [8].

Aside from gender, aging is associated with changes in walking and gait. It is pointed that in the elderly, there is a decrease in walking ability [9]. Reduced muscle strength and changes in balance, vision, and proprioception in the elderly might predispose them to the decreased walking ability [9]. With aging, there is an increase in gait and balance disorders from around 10% in persons between 60 and 69 years to 60% and above in individuals aged 80 years and above [10]. These changes can be associated with an increased risk of falls and injuries, leading further to the disability and functional limitations in the elderly. It is estimated that every third elderly person above the age of 65 experiences a fall at least once a year, and around 20% of falls need medical attention [11].

Self-assessed health and physical functioning are significant indicators of health, quality of life, and well-being among the elderly [12]. They are also consistent contributors to morbidity and mortality rates in this group [13]. The evidence prior to this study noted numerous factors influencing self-related health among the elderly, including sociodemographic characteristics, morbidity, acute and chronic diseases, functional status, mental health, and satisfaction with health services [13,14]. While women are more likely than men to seek preventive health services [15], live longer due to biological differences [16,17], and are more exposed to the risk of disabilities due to poor musculoskeletal health [18,19], a hypothesis that there are greater gender differences in physical functioning disability, not only in cognitive functioning [20], is worth exploring. A more comprehensive understanding of the gender disparity in the decline of physical functioning could enhance the development of gender-oriented active aging promotion initiatives.

Physical disability is influenced by the presence of chronic diseases, where the most prevalent were arthritis, osteoporosis, cardiovascular diseases, diabetes, hypertension, and lung diseases [21]. Furthermore, it should be stated that the presence of a functional disability is associated with a reduced quality of life, higher health care costs, and health services utilization in the elderly [22]. Moreover, Chen et al., in their study on people aged 60 years and above, pointed out that potential influencing factors of disability are gender, age, number of chronic diseases as well as educational levels, and the presence of metabolic syndrome [23].

This study aimed to assess self-perceived general health and related health characteristics among the elderly population in Serbia, with a focus on varying degrees of functional limitations, as well as to analyze the predictors of physical functioning limitations in relation to gender.

## 2. Materials and Methods

### 2.1. Study Participants

In this population-based study, 3540 elderly individuals aged above 65 years from Serbia were evaluated as a part of the third national study, “Istraživanje zdravlja stanovništva Srbije u 2013”, which was performed by the Ministry of Health of Republic of Serbia [24]. The study followed the methodology of European Health Interview Survey Wave 2 (EHIS Wave 2) [24]. The study was approved by the Institutional Review Board of the Faculty of Medicine, the University of Belgrade in Belgrade, Serbia (No: 29/III-8).

In this study, we applied a secondary and retrospective analysis of anonymous data, and survey data were obtained from the electronic database of the Institute of Public Health (IPH) of Serbia. The informed consent is not applicable.

### 2.2. Study Criteria Selection

Nationally representative probability sample was estimated according to the census of individuals, households, and apartments in the Republic of Serbia from 2011. To stratify the representative sample, the population data for Serbia according to the census from 2011 were used, and two variables were analyzed for initial stratums: region and settlement type. Four statistical regions were identified: Vojvodina, Belgrade, Sumadija and western Serbia, and southern and eastern Serbia, which were additionally divided into eight stratums regarding cities and other areas. A two-step sampling method was performed for percentage-based representation of the sample distribution at the national level. Probability proportional sampling was carried out first (670 census areas). In the second step, we performed household extraction for selected census areas (10 households and 3 additional spare households). A simple random sample without replacement was used for household selection. The study representative sample included 6500 households, of which 3540 (24.2%) had individuals aged above 65 years [24].

The inclusion criteria were private household residents living in the Republic of Serbia, while the exclusion criteria included residents of geriatric institutions and collective households, as well as those who declined to participate in the study.

### 2.3. Self-Perceived General Health

EHIS recommendations were used to evaluate self-perceived general health with a formulated question: “How is your health in general?” [25]. The answers were very good, good, fair, bad, and very bad [25]. In our study, answers were adjusted into three categories: good (very good and good categories), fair, and bad (bad and very bad).

### 2.4. Long-Lasting Health Problems

Following the EHIS recommendations, the individuals with any illness or health problems in at least 6 months were considered to have long-lasting health problems. The formulated question in this study was as follows: Do you have any longstanding illness or [longstanding] health problem? The offered answers were yes or no [25].

### 2.5. Chronic Diseases and/or Chronic Conditions

EHIS recommendations were used for defining chronic diseases and/or chronic conditions [25]. In this study, chronic diseases and/or conditions were grouped into seven groups: cardiovascular diseases; pulmonary disease; musculoskeletal diseases (MSDs); diabetes; hyperlipidemia; hypertension; and other chronic diseases (depression, cancer, urinary incontinence, kidney problems, and liver cirrhosis).

### 2.6. Difficulty in Walking Modalities

We employed a dual approach to model the four distinct difficulty levels related to the two groups of limitations of physical functioning (PF1 and 2) for male and female individuals.

The PF1 focused on walking half a kilometer on flat, level ground without the assistance of any mobility aids, and the PF2 navigated a set of 12 steps of ascent and descent. This PF1 assessment reflects an individual’s endurance and gait stability challenges, while PF2 the difficulties encountered with balance, muscle strength, and coordination.

- Model 1: inability to execute PF1,
- Model 2: some/a lot of difficulty in PF1,
- Model 3: inability to execute PF2, and
- Model 4: some/a lot of difficulty in PF2.

Initially, PF1 and 2 difficulties in walking modalities were assessed with the following questions on the individual’s functioning capacity:

1. PF1 difficulty in walking half a kilometer on level ground without the use of any aid, with the proposed question: Do you have difficulty walking half a km on level ground that would be [...] without the use of any help? The offered answers were as follows: no difficulty, some difficulty, a lot of difficulties, and cannot do at all/unable to do [25].
2. PF2 difficulty in walking up or down 12 steps with the proposed question: Do you have difficulty walking up or down 12 steps? The offered answers were as follows: no difficulty, some difficulty, a lot of difficulties, and cannot do at all/unable to do [25].

### 2.7. Statistical Analysis

Categorical variables are presented as whole numbers (n) and percentages (%). A chi-squared test was used to test the statistical significance between these variables.

For the univariate and multivariate (stepwise forward) logistic regression analysis, we formulated four models with odds ratios (ORs) and accompanied 95% confidence intervals (CIs).

- Model 1: referential value was no difficulty in performing PF1 task (0) versus unable to do (1).
- Model 2: referential value was no difficulty in performing PF1 task (0) versus some difficulty/a lot of difficulty (1).
- Model 3: referential value was no difficulty in performing PF2 task (0) versus unable to do (1).
- Model 4: referential value was no difficulty in performing PF2 task (0) versus some difficulty/a lot of difficulty (1).

The statistical significance was set at *p* < 0.05.

## 3. Results

Frequencies of self-perceived general health in both genders with regard to difficulty in walking degree are presented in Table 1. For both genders, frequencies between good, fair, and bad self-perceived general health significantly differed for those without difficulties and different degrees of difficulty in performing both PF1 and PF2 tasks (*p* < 0.001).

Males with no difficulty in performing PF1 task were about 3 times more frequently with good self-perceived general health (43.2%) than those with a bad self-perception (14.6%), while females around 1.5 times (good: 29.6% versus bad: 19.7%). For males and females unable to execute PF1 task, more than four out of five individuals had a bad self-perception (males 83.0% and females 81.4%).

For males without difficulties in performing PF2 task, good self-perception was more than 4 times more frequent (46.5%) than those with a bad self-perception (11.5%), while for females, about twice as frequent in those with good self-perception (32.3%) versus those with bad self-perception (16.1%). For males and females unable to execute PF2 task, more than four out of five individuals had a bad self-perception (males 89.2% and females 83.5%).

Frequencies of health characteristics in males regarding difficulty in walking degree are presented in Table 2. The most frequent chronic diseases were cardiovascular diseases (64.1%), followed by hypertension (58.0%) and musculoskeletal diseases (35.1%), and the least frequent were pulmonary diseases (11.3%).

There were significant differences in frequencies of evaluated health characteristics regarding the presence and degree of performing PF1 task (long-lasting health problems, pulmonary, cardiovascular, and musculoskeletal diseases, and other chronic diseases (*p* < 0.001), diabetes, hyperlipidemia, and hypertension (*p* < 0.01)). Long-lasting health problems were present in less than two-thirds of elderly males (60.3%) with no difficulty in performing PF1 task, compared to 89.6% of males who were unable to execute such a task.

There were significant differences in frequencies of evaluated health characteristics regarding the presence and degree of performing PF2 task (long-lasting health problems, cardiovascular and musculoskeletal diseases, diabetes, hypertension, and other chronic diseases (*p* < 0.001), pulmonary diseases (*p* < 0.01), and hyperlipidemia (*p* < 0.05)). Long-lasting health problems were present in less than two-thirds of elderly males (57.9%) with no difficulty in performing PF2 task, compared to 90.3% males who were unable to execute such a task.

Frequencies of health characteristics in females regarding difficulty in walking degree are presented in Table 3. The most frequent chronic diseases were cardiovascular diseases (79.0%), followed by hypertension (73.2%) and musculoskeletal diseases (54.4%), and the least frequent were pulmonary diseases (12.2%).

There were significant differences in frequencies of evaluated health characteristics regarding the presence and degree of performing PF1 task (musculoskeletal diseases (*p* < 0.001), pulmonary and cardiovascular diseases and other chronic diseases (*p* < 0.01), and long-lasting health problems and hypertension (*p* < 0.05)). Long-lasting health problems were present in more than two-thirds of elderly females (68.9%), who had no difficulty in performing the PF1 task, compared to 95.5% of females who could not execute such a task.

There were significant differences in frequencies of evaluated health characteristics regarding the presence and degree of performing PF2 task (long-lasting health problems, musculoskeletal diseases, hypertension, and other chronic diseases (*p* < 0.001), pulmonary and cardiovascular diseases (*p* < 0.01), and diabetes and hyperlipidemia (*p* < 0.05)). Long-lasting health problems were present in just below two-thirds of elderly females (64.8%), who had no difficulty in performing the PF2 task, compared to 96.0% of females who were unable to execute such tasks.

Table 4 presents univariate and multivariate (stepwise forward) logistic regression analyses of health factors associated with difficulties in performing PF1 or PF2 tasks in males.

After applying variables that were significantly associated with different degrees of disability in performing PF1 task (Model 1 and Model 2) as well as different degrees of disability in performing PF2 task (Model 3 and Model 4) from univariate into multivariate logistic regression analysis, self-perceived general health was significantly associated with all models (Models 1–4), pulmonary diseases were significantly associated with Models 2 and 4, musculoskeletal diseases were significantly associated with Models 2, 3, and 4, and other chronic diseases with Model 3 in elderly males.

Table 5 presents univariate and multivariate (stepwise forward) logistic regression analyses of health factors associated with difficulties in performing PF1 or PF2 tasks in females.

After applying variables that were significantly associated with different degrees of disability in performing PF1 task (Model 1 and Model 2) as well as different degrees of disability in performing PF2 task (Model 3 and Model 4) from univariate into multivariate logistic regression analysis, self-perceived general health and musculoskeletal diseases were significantly associated with all models (Models 1–4), long-lasting health problems was significantly associated with Models 1 and 3, and pulmonary diseases were significantly associated with Model 4 in females.

## 4. Discussion

Our findings pointed out that for elderly males, a predictor for being unable to perform PF1 task was bad self-perceived general health; for some or a lot of difficulties in performing PF1 task were bad self-perceived general health, presence of pulmonary diseases, and MSDs. In contrast, predictors for being unable to perform the PF2 task were bad self-perceived general health, MSDs, and other chronic diseases, and for some or a lot of difficulties in performing the PF2 task were bad self-perceived general health, pulmonary diseases, and MSDs. For female gender, predictors for being unable to perform PF1 task were bad self-perceived general health, long-lasting health problems, and MSDs, and for some or a lot of difficulties in performing PF1 task were bad self-perceived general health and presence of MSDs. In contrast, predictors for being unable to perform the PF2 task were bad self-perceived general health, long-lasting health problems, and MSDs, and for some/a lot of difficulties in performing the PF2 task were bad self-perceived general health, pulmonary diseases, and MSDs.

The proportion of the study population with bad self-perceived general health in both genders increased as the degree of difficulty in performing both tasks increased. In the cross-sectional study of Denche-Zamorano et al. on diabetic participants aged between 50 and 79 years, it was stated that physical inactivity increases the risk of negative self-perceived health [26]. Furthermore, in another cross-sectional study by Pereira-de-Sousa et al. on the population between 65 and 74 years of age, it was noticed that regular physical exercise is a factor related to good self-perceived health [27]. Moreover, better self-perceived health in older adults aged between 60 and 89 years was associated with higher activity levels in the study of Dostalova et al. [28]. These findings point to the importance of maintaining physical activity, and for those elderly individuals with identified difficulties in performing physical activity, individually designed interventions and adequate treatment modalities should be implemented in both home and institutionalized settings in order to achieve more favorable self-perceived general health outcomes.

This population-based study revealed that elderly males with some or a lot of difficulties executing both tasks and those who cannot execute these tasks are more likely to have a bad self-perception of health. In previous literature, it was noticed that numerous factors affect self-perceived health, including chronic diseases, muscle strength, mobility, depression, pain, group social activities, bad sleep quality, bad nutrition patterns, lower educational levels, lower income, and unemployment [1,29,30]. Therefore, in proposing interventions for the improvement of self-perception of health, a multidisciplinary approach is needed for better optimization of treatment outcomes.

As with bad self-perception of health in both genders, there is an increase in the proportion of both elderly males and elderly females with long-lasting health problems as the degree of difficulty in performing both tasks increases. Our findings pointed out that both elderly males and females with difficulties in performing the studied tasks, or participants of both genders who were unable to perform the tasks, have a high proportion of long-lasting health problems. In the study of Ostir et al., it was suggested that morbidity, disability, and mortality are interrelated [31]. Rather than prevent chronic conditions, early diagnosis and enhanced treatment and management are suggested as important contributing factors for improving upper and lower body functioning in older individuals [32]. These findings stress the complexity of the interaction between chronic health conditions and disability, thus implying the necessity of preventive strategies and measures along with treatment intervention promotions in populations who are at risk, particularly elderly individuals. Furthermore, it was observed that sex disparities are present in disease prevalence, pathophysiology, clinical manifestation, and treatment response [33]. Additionally, physical activity patterns were shown to differ between genders with different chronic diseases [34]. Therefore, individually designed interventions bearing in mind gender in the elderly, on primary, secondary, and tertiary levels, are advised.

The long-lasting health problems that emerged as predictors in females from this study for difficulty in performing both tasks might be explained to a certain degree by the fact that females have a higher risk for multimorbidity, obesity, and physical disability than males [35]. It is worth mentioning that individual psychological, social, environmental, and demographic factors influence physical activity levels in various populations; for example, in terms of gender roles, females and males might be affected differently in cases where living with children [36]. Therefore, all of these dimensions should be taken into consideration when planning and implementing strategies and interventions for optimizing and improving physical functionality in both genders.

In our study, as the degree of difficulty in performing both tasks increases, the proportion of present pulmonary diseases increases in both genders. Our findings revealed that elderly males with physical functioning impairments have a slightly higher presence of pulmonary diseases than elderly females. In the elderly with chronic obstructive pulmonary disease (COPD), it was noticed that an increase in fatigue increases disability levels [37]. Furthermore, in the study of Woo, it was stated that dyspnea, fatigue, and physical activity in patients with COPD are interrelated [38]. In addition to this, it should be noted that in the elderly with COPD, other conditions can be present, such as depression and anxiety, and in COPD patients, particularly those with hypoxemia, there is an increased risk of cognitive impairment [39]. All of these might have an effect on physical functioning, physical performance, and levels of disability, leading to the necessity of a structural multidisciplinary individual approach in the creation, implementation, and monitoring of preventive and treatment interventions in this group of elderly individuals. In line with this, there are numerous beneficial effects of physical training in COPD patients, such as maximal exercise, walking distance, functional performance improvement, and improvement in the quality of life [40].

Considering MSDs, an increase in the degree of difficulty in performing both tasks from this study increases the proportion of MSDs in both genders. Musculoskeletal diseases are associated with disability, pain, mobility disorders as well as increased risk for falls and fractures [41,42]. The levels of physical activity in people with MSD are considered to be low [43]. Still, in the systematic review and meta-analysis of Gwinnutt et al., it was stated that exercise interventions in people with rheumatic and musculoskeletal diseases lead to the improvement in pain and function [44], leading to the necessity of implementation and optimization of individually designed rehabilitation interventions bearing in mind the presence of comorbidities as well as cognitive and physio-biological changes in elderly of both genders for better physical functioning and health improvement. To achieve better and optimal outcomes and responses to physical activity, this population’s barriers and facilitators should be considered. These barriers and facilitators can be grouped into physical and psychological capabilities, physical and social opportunities, and reflective and automatic motivation [45]. The potential facilitators in people with musculoskeletal diseases might include good health expectancy, exercise enjoyment, losing weight, and being fit [43], while potential barriers might include fatigue, lack of motivation, and pain [43].

As with pulmonary diseases and MSDs, as the degree of difficulty in both tasks performance from our study increases, the proportion of other chronic diseases also increases. The presence of other chronic diseases emerged as a predictor in elderly males who were unable to walk up or down 12 steps.

### 4.1. Limitations

The implications of the study findings must be interpreted with caution, as they rely on self-reported data, which is vulnerable to various forms of bias. This includes the ability to comprehend questions without confusion about their meanings or guessing, the capacity to accurately assess their conditions, and the tendency to conform to socially acceptable responses or favorable social norms, influenced by their interest or desire to complete the survey quickly [46]. To minimize information bias, self-reported data should be corroborated by objective functional tests of study respondents. Ideally, clinical measurement could be included in parallel to the national health survey in the future. Moreover, it could reveal specific pathologies besides the main reported chronic conditions. Additionally, the inclusion of psychosocial factors and their impact on mobility in the elderly are recommended for future investigations.

Although the study is population-based, the sample is large and representative, and the study instruments were internationally verified, all in favor of providing a solid foundation for generating sound evidence on elderly physical ability, these conclusions may not be fully justifiable in vastly different contexts. Potential selection bias could be that the study did not include elderly individuals from geriatric institutions and collective households.

This study design is cross-sectional, which limits the investigation of causal relationships but allows the exploration of a hypothesis regarding gender differences in physical functioning limitations.

### 4.2. Policy and Practice Implications

The models provide a credible framework for future research on gender issues within the elderly population, especially those who support healthy and active aging. The study indicates that successful policies and practices for gender-specific medicine should emphasize self-assessment, which reveals notable gender differences regarding perceived impacts on physical activity. By recognizing gender inequalities in physical functioning, healthcare providers can tailor interventions to address the unique needs and challenges each gender faces. This gender-focused approach may improve disease management by encouraging older individuals to pursue promotional interventions that meet their expectations regarding physical functioning improvement and active aging. In addition to this, given that various conditions along with aging and gender can affect mobility in the elderly, it is important to include multidisciplinary, interdisciplinary, and transdisciplinary approaches in interventional programs creation, their implementation, and efficacy monitoring for optimal functional outcomes.

## 5. Conclusions

Bad self-perceived general health and pulmonary and musculoskeletal diseases were predictors of functional disability in both genders of elderly individuals. In contrast, long-lasting health problems were predictors of functional disability in females and other chronic diseases predictors in elderly males.

A better understanding of the specific health dimensions and self-perceived general health in elderly males and females, along with their specific physiological, biological, and psycho-social differences, is of particular importance in establishing and promoting strategies and effective gender-oriented measures related to the prevention of further functional deterioration and optimizing interventions to increase participation, physical functioning, and performance in the community.

## Figures and Tables

**Table 1:** Frequencies of self-perceived general health in both genders with regard to difficulty in walking degree.

Self-Perceived General Health, n (%)	No Difficulty	Some Difficulty	A Lot of Difficulty	Unable to Do	Total	*p*
Males						
PF1 task						
Good	387 (43.2)	49 (16.6)	14 (6.1)	3 (2.8)	453 (29.7)	<0.001
Fair	377 (42.1)	130 (43.9)	60 (26.1)	15 (14.2)	582 (38.1)	<0.001
Bad	131 (14.6)	117 (39.5)	156 (67.8)	88 (83.0)	492 (32.2)	<0.001
*p*	<0.001	<0.001	<0.001	<0.001	<0.001	
PF2 task						
Good	379 (46.5)	53 (15.3)	19 (7.0)	2 (2.2)	453 (29.7)	<0.001
Fair	342 (42.0)	166 (48.0)	66 (24.2)	8 (8.6)	582 (38.1)	<0.001
Bad	94 (11.5)	127 (36.7)	188 (68.9)	83 (89.2)	492 (32.2)	<0.001
*p*	<0.001	<0.001	<0.001	<0.001	<0.05	
Females						
PF1 task						
Good	221 (29.6)	71 (13.0)	27 (6.3)	17 (5.8)	336 (16.7)	<0.001
Fair	379 (50.7)	239 (43.8)	97 (22.7)	37 (12.7)	752 (37.4)	<0.001
Bad	147 (19.7)	236 (43.2)	304 (71.0)	237 (81.4)	924 (45.9)	<0.001
*p*	<0.001	<0.001	<0.001	<0.001	<0.001	
PF2 task						
Good	190 (32.3)	97 (15.9)	39 (6.9)	10 (4.0)	336 (16.7)	<0.001
Fair	304 (51.6)	280 (46.0)	137 (24.2)	31 (12.4)	752 (37.4)	<0.001
Bad	95 (16.1)	232 (38.1)	389 (68.8)	208 (83.5)	924 (45.9)	<0.001
*p*	<0.001	<0.001	<0.001	<0.001	<0.001	

*p*—Chi-squared test.

**Table 2:** Frequencies of health characteristics in males with regard to difficulty in walking degree.

Health Characteristics, n (%)	No Difficulty	Some Difficulty	A Lot of Difficulty	Unable to Do	Total	*p*
PF1 task						
Long-lasting health problems,	539 (60.3)	227 (76.7)	212 (92.6)	95 (89.6)	1073 (70.4)	<0.001
Chronic diseases and/or chronic conditions						
Pulmonary diseases	57 (6.4)	49 (16.6)	47 (20.5)	19 (17.9)	172 (11.3)	<0.001
Cardiovascular diseases	519 (57.9)	205 (69.3)	170 (73.9)	85 (80.2)	979 (64.1)	<0.001
Musculoskeletal diseases	238(26.6)	120 (40.5)	120 (52.4)	58 (54.7)	536 (35.1)	<0.001
Diabetes	121 (13.5)	49 (16.8)	65 (28.4)	30 (28.6)	265 (17.4)	<0.01
Hyperlipidemia	135 (15.3)	47 (16.2)	43 (19.8)	24 (23.1)	249 (16.7)	<0.01
Other chronic diseases	201 (22.4)	101 (34.1)	100 (43.7)	57 (53.8)	459 (30.1)	<0.001
Hypertension	479 (53.6)	183 (61.8)	149 (64.8)	73 (70.2)	884 (58.0)	<0.01
*p*	<0.001	<0.001	<0.001	<0.001	<0.001	
PF2 task						
Long-lasting health problems	472 (57.9)	265 (76.8)	252 (92.6)	84 (90.3)	1073 (70.4)	<0.001
Chronic diseases and/or chronic conditions						
Pulmonary diseases	47 (5.8)	52 (15.1)	55 (20.2)	18 (19.4)	172 (11.3)	<0.01
Cardiovascular diseases	456 (55.9)	241 (59.7)	206 (75.5)	76 (81.7)	979 (64.1)	<0.001
Musculoskeletal diseases	202 (24.8)	148 (42.8)	137 (50.4)	49 (52.7)	536 (35.1)	<0.001
Diabetes	104 (12.8)	59 (17.1)	73 (26.8)	29 (31.5)	265 (17.4)	<0.001
Hyperlipidemia	119 (14.8)	58 (17.5)	50 (18.9)	22 (24.4)	249 (16.7)	<0.05
Other chronic diseases	176 (21.6)	111 (32.1)	118 (43.4)	54 (58.1)	459 (30.1)	<0.001
Hypertension	429 (52.7)	212 (61.3)	178 (65.2)	65 (71.4)	884 (58.0)	<0.001
*p*	<0.001	<0.001	<0.001	<0.001	<0.001	

*p*—Chi-squared test.

**Table 3:** Frequencies of health characteristics in females with regard to difficulty in walking degree.

Health Characteristics, n (%)	No Difficulty	Some Difficulty	A Lot of Difficulty	Unable to Do	Total	*p*
PF1 task						
Long-lasting health problems	513 (68.9)	434 (79.9)	382 (89.9)	277 (95.5)	1606 (80.2)	<0.05
Chronic diseases and/or chronic conditions						
Pulmonary diseases	52 (7.0)	76 (13.9)	68 (16.0)	49 (17.1)	245 (12.2)	<0.01
Cardiovascular diseases	551 (73.8)	425 (77.8)	367 (85.7)	246 (84.8)	1589 (79.0)	<0.01
Musculoskeletal diseases	287 (38.4)	306 (56.0)	286 (67.0)	214 (74.0)	1093 (54.4)	<0.001
Diabetes	127 (17.0)	107 (19.7)	84 (19.8)	56 (19.9)	374 (18.7)	> 0.05
Hyperlipidemia	194 (26.5)	128 (24.3)	112 (27.6)	68 (25.6)	502 (26.0)	>0.05
Other chronic diseases	214 (28.6)	189 (34.6)	174 (40.7)	146 (50.3)	723 (36.0)	<0.01
Hypertension	512 (68.7)	400 (73.5)	334 (78.8)	218 (76.0)	1464 (73.2)	<0.05
*p*	<0.001	<0.001	<0.001	<0.001	<0.001	
PF2 task						
Long-lasting health problems	381 (64.8)	481 (79.5)	507 (90.1)	237 (96.0)	1606 (80.2)	<0.001
Chronic diseases and/or chronic conditions						
Pulmonary diseases	32 (5.4)	75 (12.3)	94 (16.7)	44 (17.9)	245 (12.2)	<0.01
Cardiovascular diseases	417 (70.8)	480 (78.8)	488 (86.4)	204 (82.3)	1589 (79.0)	<0.01
Musculoskeletal diseases	206 (35.0)	300 (49.3)	401 (71.0)	186 (75.3)	1093 (54.4)	<0.001
Diabetes	87 (14.8)	131 (21.6)	106 (18.9)	50 (20.7)	374 (18.7)	<0.05
Hyperlipidemia	146 (25.3)	158 (26.8)	151 (28.4)	47 (20.3)	502 (26.0)	<0.05
Other chronic diseases	155 (26.3)	202 (33.2)	244 (43.3)	122 (49.2)	723 (36.0)	<0.001
Hypertension	390 (66.2)	436 (72.4)	458 (81.5)	180 (72.9)	1464 (73.2)	<0.001
*p*	<0.001	<0.001	<0.001	<0.001	<0.001	

*p*—Chi-squared test.

**Table 4:** Health factors associated with difficulties in performing PF1 or PF2 tasks in males.

Variables	Model 1 OR (95% CI)	Model 2 OR (95% CI)	Model 3 OR (95% CI)	Model 4 OR (95% CI)
Univariate logistic regression				
Self-perceived general health	13.406 *** (8.363–21.490)	3.645 *** (3.079–4.314)	26.164 *** (14.138–48.422)	4.241 *** (3.573–5.034)
Long-lasting health problems	5.704 *** (3.013–10.801)	3.362 *** (2.574–4.392)	6.802 *** (3.373–13.716)	3.757 *** (2.910–4.850)
Pulmonary diseases	3.218 *** (1.831–5.658)	3.302 *** (2.332–4.675)	3.932 *** (2.174–7.113)	3.433 *** (2.393–4.924)
Cardiovascular diseases	2.948 *** (1.796–4.839)	1.804 *** (1.432–2.272)	3.539 *** (2.055–6.096)	2.052 *** (1.641–2.566)
Musculoskeletal diseases	3.322 *** (2.204–5.006)	2.325 *** (1.853–2.917)	3.363*** (2.172–5.205)	2.597 *** (2.075–3.250)
Diabetes	2.555 *** (1.606–4.067)	1.770 *** (1.334–2.347)	3.143 *** (1.934–5.107)	1.852 *** (1.397–2.454)
Hyperlipidemia	1.658 (1.014–2.710)	1.191 (0.889–1.596)	1.868 * (1.112–3.137)	1.276 (0.959–1.697)
Other chronic diseases	4.028 *** (2.666–6.087)	2.145 *** (1.694–2.716)	5.043 *** (3.234–7.864)	2.141 *** (1.695–2.703)
Hypertension	2.045 ** (1.317–3.176)	1.483 *** (1.189–1.849)	2.249 ** (1.399–3.618)	1.528 *** (1.235–1.892)
Multivariate (stepwise forward) logistic regression				
Self-perceived general health	8.639 *** (5.236–14.254)	2.759 *** (2.303–3.305)	24.290 *** (12.634–46.699)	3.256 *** (2.714–3.906)
Long-lasting health problems	-	-	-	-
Pulmonary diseases	-	2.036 *** (1.371–3.023)	-	1.976 ** (1.301–3.000)
Cardiovascular diseases	-	-	-	-
Musculoskeletal diseases	-	1.397 * (1.073–1.818)	1.954 * (1.102–3.464)	1.531 ** (1.174–1.996)
Diabetes	-	-	-	-
Hyperlipidemia	-	-	-	-
Other chronic diseases	-	-	2.215 ** (1.258–3.903)	-
Hypertension	-	-	-	-

OR—odds ratio; CI—confidence interval; * *p* < 0.05; ** *p* < 0.01; *** *p* < 0.001.

**Table 5:** Health factors associated with difficulties in performing PF1 or PF2 tasks in females.

Variables	Model 1 OR (95% CI)	Model 2 OR (95% CI)	Model 3 OR (95% CI)	Model 4 OR (95% CI)
Univariate logistic regression				
Self-perceived general health	8.197 *** (6.139–10.944)	3.045 *** (2.625–3.533)	12.325 *** (8.694–17.472)	3.072 *** (2.643–3.571)
Long-lasting health problems	9.636 *** (5.409–17.166)	2.428 *** (1.924–3.063)	12.876 *** (6.689–24.788)	2.982 *** (2.364–3.761)
Pulmonary diseases	2.752 *** (1.813–4.176)	2.324 *** (1.666–3.242)	3.791 *** (2.339–6.146)	2.936 *** (1.984–4.344)
Cardiovascular diseases	1.999 *** (1.388–2.850)	1.548 *** (1.231–1.947)	1.912 ** (1.320–2.772)	1.938 *** (1.536–2.446)
Musculoskeletal diseases	4.573 *** (3.383–6.183)	2.490 *** (2.048–3.029)	5.669 *** (4.055–7.926)	2.761 *** (2.248–3.392)
Diabetes	1.210 (0.853–1.715)	1.200 (0.936–1.538)	1.511 * (1.027–2.221)	1.470 ** (1.124–1.924)
Hyperlipidemia	0.951 (0.690–1.310)	0.960 (0.770–1.197)	0.754 (0.520–1.093)	1.123 (0.894–1.412)
Other chronic diseases	2.525 *** (1.909–3.340)	1.482 *** (1.207–1.819)	2.711 *** (1.990–3.693)	1.718 *** (1.381–2.136)
Hypertension	1.438 * (1.053–1.964)	1.427 ** (1.153–1.767)	1.371 * (0.987–1.904)	1.690 *** (1.358–2.102)
Multivariate (stepwise forward) logistic regression				
Self-perceived general health	3.569 *** (2.589–4.919)	2.277 *** (1.940–2.671)	5.090 *** (3.498–7.406)	2.152 *** (1.815–2.553)
Long-lasting health problems	2.867 *** (1.416–5.805)	-	3.602 ** (1.677–7.733)	-
Pulmonary diseases	-	-	-	1.756 ** (1.151–2.679)
Cardiovascular diseases	-	-	-	-
Musculoskeletal diseases	1.537 * (1.039–2.273)	1.410 ** (1.126–1.765)	1.739 * (1.109–2.728)	1.483 ** (1.171–1.879)
Diabetes	-	-	-	-
Hyperlipidemia	-	-	-	-
Other chronic diseases	-	-	-	-
Hypertension	-	-	-	-

OR—odds ratio; CI—confidence interval; * *p* < 0.05; ** *p* < 0.01; *** *p* < 0.001.

## Data Availability

Data are available upon reasonable request from the last author Milena Kostadinovic.
